# PP89 Artificial Intelligence And Health Technology Assessment: Playing Catch-Up

**DOI:** 10.1017/S0266462324002575

**Published:** 2025-01-07

**Authors:** Clare England, Jenni Washington

## Abstract

**Introduction:**

The use of artificial intelligence (AI) in health care has the potential to improve clinical and patient outcomes and to reduce rising costs. There is an exponential increase in health technologies that use AI. We present a health technology assessment (HTA) case study demonstrating that the rapid rise in publications presents challenges for HTA bodies seeking to provide robust, timely assessments.

**Methods:**

We conducted an HTA of AI-assisted endoscopy in the detection and characterization of lower gastrointestinal (GI) cancer. Searches were conducted up to October 2023. Unusually, the search targeted only the intervention: artificial intelligence and lower-GI-tract endoscopy (including colonoscopy, proctoscopy, etc.). The search strategy was peer reviewed. MEDLINE, Embase, KSR Evidence, CINAHL, Cochrane Library, and the INAHTA HTA database were searched, as well as ongoing trial registers and key websites. A date limit of 2010 onwards was applied, as Xbox Kinect launched in 2010 and was the first mainstream device used for healthcare imaging.

**Results:**

Two network meta-analyses, 15 meta-analyses, one systematic review of meta-analyses, and three systematic reviews published since 2020 were identified. One review conducted searches to January 2020, identifying three randomized controlled trials (RCTs); a review that searched up to February 2023 identified 21 RCTs. There was substantial overlap regarding included primary studies, but not all reviews included the same outcomes. An additional seven RCTs were published in 2023. We also identified 12 cohort studies published between 2021 and 2023. We prioritized higher quality meta-analyses of clinical RCTs to include all outcomes of interest and updated the meta-analyses for primary outcomes.

**Conclusions:**

This case study of an HTA of an AI-related technology demonstrates how rapidly the field is moving. It is necessary to use well targeted but not overly exclusive search strategies, limit by date, and prioritize inclusion of identified evidence according to quality and availability of outcomes. Time should be allowed to update existing meta-analyses.

